# [μ-Bis(5,7-dimethyl-1,8-naphthyridin-2-yl)diazene]bis­[difluoridoboron(III)]

**DOI:** 10.1107/S1600536809022491

**Published:** 2009-06-24

**Authors:** Xin-Sheng Li, Juan Mo, Li Yuan, Jian-Hua Liu, Su-Mei Zhang

**Affiliations:** a College of Animal Husbandry and Veterinary Studies, Henan Agricultural University, Zhengzhou, Henan Province 450002, People’s Republic of China

## Abstract

In the title compound, C_20_H_18_B_2_F_4_N_6_, the bis­(5,7-dimethyl-1,8-naphthyridin-2-yl)diazene molecule is bis­ected by a symmetry centre midway between the central N atoms of the diazene group. Each of the symmetry-related halves of the molecule binds to a B atom through an *N*,*N*′-bite. Two terminal F ions complete the distorted BN_2_F_2_ tetra­hedral geometry around each B atom. The BF_2_ plane is almost perpendicular to the boron–naphthyridine ring plane, with a dihedral angle of 87.8 (2)°. The main inter­actions in the crystal structure are some C—H⋯F hydrogen bonds and π–π contacts between 1,8-naphthyridine rings [centroid–centroid distance = 4.005 (1) Å].

## Related literature

For 1,8-naphthyridine deriatives, see: Gavrilova & Bosnich (2004 ▶); Goswami & Mukherjee (1997 ▶); Nakatani *et al.* (2000 ▶).
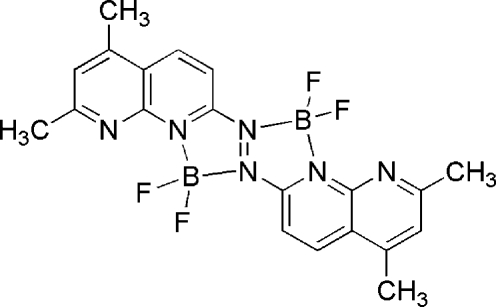


## Experimental

### 

#### Crystal data

C_20_H_18_B_2_F_4_N_6_
*M* *_r_* = 440.02
Orthorhombic, 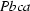

*a* = 8.5379 (17) Å
*b* = 14.696 (3) Å
*c* = 15.467 (3) Å
*V* = 1940.6 (7) Å^3^
*Z* = 4
Mo *K*α radiation
μ = 0.12 mm^−1^
*T* = 272 K
0.10 × 0.08 × 0.06 mm

#### Data collection

Bruker SMART CCD area-detector diffractometer
Absorption correction: multi-scan (*SADABS*; Sheldrick, 2003 ▶) *T*_min_ = 0.985, *T*_max_ = 0.994
14645 measured reflections
2221 independent reflections
1714 reflections with *I* > 2σ(*I*)
*R*_int_ = 0.058

#### Refinement

*R*[*F* ^2^ > 2σ(*F* ^2^)] = 0.045
*wR*(*F* ^2^) = 0.127
*S* = 1.07
2221 reflections
148 parameters
H-atom parameters constrained
Δρ_max_ = 0.32 e Å^−3^
Δρ_min_ = −0.24 e Å^−3^

### 

Data collection: *SMART* (Bruker, 2000 ▶); cell refinement: *SAINT* (Bruker, 2000 ▶); data reduction: *SAINT*; program(s) used to solve structure: *SHELXS97* (Sheldrick, 2008 ▶); program(s) used to refine structure: *SHELXL97* (Sheldrick, 2008 ▶); molecular graphics: *SHELXTL* (Sheldrick, 2008 ▶); software used to prepare material for publication: *SHELXTL*.

## Supplementary Material

Crystal structure: contains datablocks global, I. DOI: 10.1107/S1600536809022491/bg2262sup1.cif

Structure factors: contains datablocks I. DOI: 10.1107/S1600536809022491/bg2262Isup2.hkl

Additional supplementary materials:  crystallographic information; 3D view; checkCIF report

## Figures and Tables

**Table 1:** Hydrogen-bond geometry (Å, °)

*D*—H⋯*A*	*D*—H	H⋯*A*	*D*⋯*A*	*D*—H⋯*A*
C1—H1*C*⋯F1^i^	0.96	2.54	3.291 (2)	135
C8—H8⋯F1^ii^	0.93	2.48	3.2434 (19)	140

Symmetry codes: (i) 
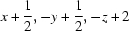
; (ii) 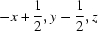.

**Fig. 1.**
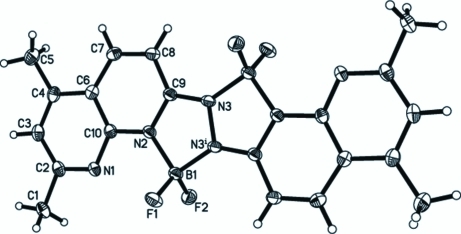
Molecular structure of the title compound showing the atom-numbering scheme and displacement ellipsoids drawn at the 40% probability level. (Symmetry code: -x, -y, 2 - z).

**Fig. 2.**
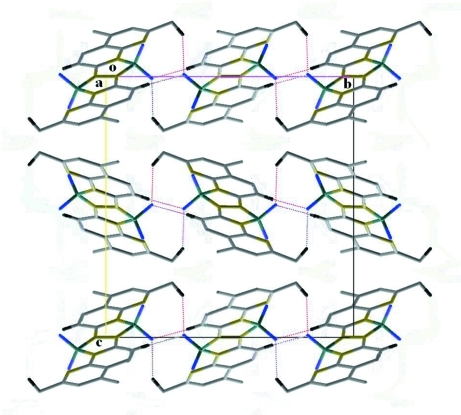
The crystal packing of the title compound, viewed along the a axis. Hydrogen bonds are shown as dashed lines.

## supplementary crystallographic information

### Comment

The deriatives of 1,8-naphthyridine have been widely utilized as mono-nucleating and dinucleating ligands in coordination chemistry or as antimycobacterial and antimicrobial agents (Gavrilova & Bosnich, 2004; Goswami *et al.*, 1997; Nakatani *et al.*, 2000). Here, we report the crystal structure of the centrosymmetric dinuclear title compound, C_20_H_18_B_2_F_4_N_6_, where the di-5,7-dimethyl-1,8-naphthyl-2,2'-diazene (ddnd) ligand is halved by a symmetry centre midway the central nitrogens in the  diazene group, and where each boron atom is coordinated by a  N,N' bite of the  ddnd ligand and two terminal F ions. The result is a distorted BN_2_F_2_ tetrahedral geometry, with a N—B—N bite angle of 94.35 (11)°. The compound skeleton is formed by four conjugated heterocyclic rings which are nearly coplanar; the least-squares plane through B1,N1,N2,C2->C10 has a mean deviation of 0.02 Å. The BF_2_ plane is almost perpendicular to the boron-nathphyridine ring plane, with a dihedral angle of 92.2 (2) °.

There are two main C—H···F hydrogen bonding interactions involving F1 as an acceptor and two C—H donor sets, C1—H1C, C8—H8. There is also a π-π  stacking interaction between adjacent parallel naphthyridyl rings: with a closest C—C distance of  3.499 (1) Å and a centroid to centroid distance of 4.005 (1) Å. Via these interactions (with H-bonding mainly in the (100) plane and π-π  stacking along the [100] direction ) the compound  forms  a three-dimensional network structure as shown in Fig. 2.

### Experimental

A cold solution of 7-amino-2,4-dimethyl-1,8naphthyridine (2 g) in water (100 mL) was added dropwise to 150 ml of a 10% NaOCl solution. The mixture was stirred at 0 degree and a dark green precipitate formed. Filtration was performed a few minutes after the end of addition, and the aqueous phase and the precipitate were extracted with diethyl ether. The ether phases were gathered, dried on MgSO_4_, and evaporated. The crude product was purified by chromatography on Al_2_O_3_ (eluent: acetone/hexane 1/10). Recrystallization in water yielded ddnd as green product. Yield: 70%.

The ddnd (0.34 g, 1 mmol) ligand was dissolved in 50 ml newly dry dichloromethane and then treated with triethylamine (3 ml) and boron trifluoride etherate (3 ml). After stirring for 30 min, the solution was washed with water, dried over Na_2_SO_4_, and concentrated at reduced pressure. Single crystals of (I) suitable for an X-ray study were obtained by slow evaporation of an CHCl_3_/hexane solution (50% *v*/*v*) over a period of one month.

### Refinement

All hydrogen atoms were generated geometrically (C—H bond lengths of methyl group fixed at 0.96 Å, C—H bond lengths of naphthyridine fixed at 0.93 Å), assigned appropriated isotropic thermal parameters, *U*_iso_(H) = 1.2*U*_eq_(C).

### Crystal data

**Table:**

C_20_H_18_B_2_F_4_N_6_	*F*(000) = 904
*M**_r_* = 440.02	*D*_x_ = 1.506 Mg m^−^^3^
Orthorhombic, *P**b**c**a*	Mo *K*α radiation, λ = 0.71073 Å
Hall symbol: -P 2ac 2ab	Cell parameters from 3723 reflections
*a* = 8.5379 (17) Å	θ = 3.1–27.5°
*b* = 14.696 (3) Å	µ = 0.12 mm^−^^1^
*c* = 15.467 (3) Å	*T* = 272 K
*V* = 1940.6 (7) Å^3^	Block, brown
*Z* = 4	0.10 × 0.08 × 0.06 mm

### Data collection

**Table:**

Bruker SMART CCD area-detector diffractometer	2221 independent reflections
Radiation source: fine-focus sealed tube	1714 reflections with *I* > 2σ(*I*)
graphite	*R*_int_ = 0.058
φ and ω scans	θ_max_ = 27.5°, θ_min_ = 3.1°
Absorption correction: multi-scan (*SADABS*; Sheldrick, 2003)	*h* = −9→11
*T*_min_ = 0.985, *T*_max_ = 0.994	*k* = −19→15
14645 measured reflections	*l* = −20→19

### Refinement

**Table:**

Refinement on *F*^2^	Secondary atom site location: difference Fourier map
Least-squares matrix: full	Hydrogen site location: inferred from neighbouring sites
*R*[*F*^2^ > 2σ(*F*^2^)] = 0.045	H-atom parameters constrained
*wR*(*F*^2^) = 0.127	*w* = 1/[σ^2^(*F*_o_^2^) + (0.0655*P*)^2^ + 0.3984*P*] where *P* = (*F*_o_^2^ + 2*F*_c_^2^)/3
*S* = 1.07	(Δ/σ)_max_ = 0.002
2221 reflections	Δρ_max_ = 0.32 e Å^−^^3^
148 parameters	Δρ_min_ = −0.24 e Å^−^^3^
0 restraints	Extinction correction: *SHELXL*, Fc^*^=kFc[1+0.001xFc^2^λ^3^/sin(2θ)]^-1/4^
Primary atom site location: structure-invariant direct methods	Extinction coefficient: 0.012 (2)

### Special details

**Table:**

Geometry. All e.s.d.'s (except the e.s.d. in the dihedral angle between two l.s. planes) are estimated using the full covariance matrix. The cell e.s.d.'s are taken into account individually in the estimation of e.s.d.'s in distances, angles and torsion angles; correlations between e.s.d.'s in cell parameters are only used when they are defined by crystal symmetry. An approximate (isotropic) treatment of cell e.s.d.'s is used for estimating e.s.d.'s involving l.s. planes.
Refinement. Refinement of *F*^2^ against ALL reflections. The weighted *R*-factor *wR* and goodness of fit *S* are based on *F*^2^, conventional *R*-factors *R* are based on *F*, with *F* set to zero for negative *F*^2^. The threshold expression of *F*^2^ > σ(*F*^2^) is used only for calculating *R*-factors(gt) *etc*. and is not relevant to the choice of reflections for refinement. *R*-factors based on *F*^2^ are statistically about twice as large as those based on *F*, and *R*- factors based on ALL data will be even larger.

### Fractional atomic coordinates and isotropic or equivalent isotropic displacement parameters (Å^2^)

**Table:**

	*x*	*y*	*z*	*U*_iso_*/*U*_eq_	
F1	0.11305 (12)	0.18492 (6)	1.00731 (7)	0.0319 (3)	
F2	−0.00219 (12)	0.14892 (7)	0.87999 (7)	0.0323 (3)	
N1	0.36490 (16)	0.15195 (8)	0.86608 (9)	0.0227 (3)	
N2	0.20684 (15)	0.04677 (8)	0.93336 (9)	0.0221 (3)	
N3	0.03539 (17)	−0.04321 (8)	1.00022 (10)	0.0260 (4)	
B1	0.0693 (2)	0.11566 (11)	0.95247 (13)	0.0243 (4)	
C1	0.5141 (2)	0.26721 (11)	0.79215 (12)	0.0319 (4)	
H1A	0.4310	0.2784	0.7517	0.048*	
H1B	0.6132	0.2741	0.7635	0.048*	
H1C	0.5071	0.3099	0.8390	0.048*	
C2	0.50004 (19)	0.17214 (10)	0.82689 (11)	0.0254 (4)	
C3	0.6203 (2)	0.10902 (11)	0.81609 (12)	0.0279 (4)	
H3	0.7136	0.1272	0.7903	0.033*	
C4	0.60339 (19)	0.02021 (11)	0.84293 (11)	0.0264 (4)	
C5	0.7318 (2)	−0.04743 (12)	0.82761 (13)	0.0345 (4)	
H5A	0.7073	−0.0835	0.7776	0.052*	
H5B	0.7418	−0.0863	0.8772	0.052*	
H5C	0.8287	−0.0158	0.8182	0.052*	
C6	0.46063 (19)	−0.00410 (10)	0.88339 (11)	0.0235 (4)	
C7	0.4213 (2)	−0.09362 (10)	0.91393 (11)	0.0252 (4)	
H7	0.4938	−0.1404	0.9078	0.030*	
C8	0.2814 (2)	−0.11140 (10)	0.95139 (11)	0.0245 (4)	
H8	0.2559	−0.1699	0.9696	0.029*	
C9	0.17464 (19)	−0.03837 (10)	0.96207 (11)	0.0226 (4)	
C10	0.34843 (19)	0.06558 (10)	0.89359 (11)	0.0217 (4)	

### Atomic displacement parameters (Å^2^)

**Table:**

	*U*^11^	*U*^22^	*U*^33^	*U*^12^	*U*^13^	*U*^23^
F1	0.0394 (6)	0.0203 (5)	0.0361 (7)	−0.0040 (4)	0.0109 (5)	−0.0022 (4)
F2	0.0286 (6)	0.0336 (5)	0.0347 (7)	0.0027 (4)	0.0020 (5)	0.0125 (4)
N1	0.0224 (7)	0.0230 (6)	0.0227 (8)	−0.0048 (5)	0.0010 (6)	0.0018 (5)
N2	0.0233 (7)	0.0184 (6)	0.0245 (8)	−0.0016 (5)	0.0034 (6)	0.0027 (5)
N3	0.0261 (7)	0.0154 (6)	0.0366 (9)	0.0022 (5)	0.0087 (6)	0.0039 (5)
B1	0.0260 (10)	0.0187 (8)	0.0283 (11)	−0.0004 (6)	0.0047 (8)	0.0029 (7)
C1	0.0326 (10)	0.0296 (8)	0.0335 (10)	−0.0099 (7)	0.0043 (8)	0.0039 (7)
C2	0.0269 (9)	0.0285 (8)	0.0207 (9)	−0.0076 (6)	−0.0011 (7)	0.0000 (6)
C3	0.0224 (9)	0.0368 (9)	0.0245 (9)	−0.0059 (7)	0.0019 (7)	0.0007 (7)
C4	0.0224 (8)	0.0345 (8)	0.0223 (9)	−0.0009 (6)	−0.0015 (7)	−0.0018 (7)
C5	0.0274 (9)	0.0419 (10)	0.0341 (11)	0.0045 (7)	0.0049 (8)	−0.0006 (8)
C6	0.0224 (8)	0.0282 (8)	0.0198 (9)	0.0010 (6)	0.0001 (7)	−0.0007 (6)
C7	0.0272 (9)	0.0251 (7)	0.0234 (9)	0.0044 (6)	0.0000 (7)	−0.0013 (6)
C8	0.0279 (9)	0.0193 (7)	0.0262 (9)	0.0017 (6)	0.0012 (7)	0.0009 (6)
C9	0.0245 (8)	0.0190 (7)	0.0244 (9)	−0.0005 (6)	0.0027 (7)	0.0023 (6)
C10	0.0221 (8)	0.0232 (7)	0.0197 (8)	−0.0028 (6)	0.0008 (7)	0.0001 (6)

### Geometric parameters (Å, °)

**Table:**

F1—B1	1.377 (2)	C2—C3	1.394 (2)
F2—B1	1.367 (2)	C3—C4	1.377 (2)
N1—C2	1.337 (2)	C3—H3	0.9300
N1—C10	1.3461 (19)	C4—C6	1.416 (2)
N2—C9	1.3559 (18)	C4—C5	1.499 (2)
N2—C10	1.384 (2)	C5—H5A	0.9600
N2—B1	1.579 (2)	C5—H5B	0.9600
N3—C9	1.329 (2)	C5—H5C	0.9600
N3—N3^i^	1.407 (2)	C6—C10	1.411 (2)
N3—B1^i^	1.571 (2)	C6—C7	1.438 (2)
B1—N3^i^	1.571 (2)	C7—C8	1.353 (2)
C1—C2	1.502 (2)	C7—H7	0.9300
C1—H1A	0.9600	C8—C9	1.417 (2)
C1—H1B	0.9600	C8—H8	0.9300
C1—H1C	0.9600		
			
C2—N1—C10	116.28 (14)	C3—C4—C6	117.55 (15)
C9—N2—C10	120.45 (13)	C3—C4—C5	120.28 (16)
C9—N2—B1	112.31 (13)	C6—C4—C5	122.16 (15)
C10—N2—B1	127.23 (12)	C4—C5—H5A	109.5
C9—N3—N3^i^	109.50 (15)	C4—C5—H5B	109.5
C9—N3—B1^i^	138.76 (12)	H5A—C5—H5B	109.5
N3^i^—N3—B1^i^	111.70 (16)	C4—C5—H5C	109.5
F2—B1—F1	111.22 (13)	H5A—C5—H5C	109.5
F2—B1—N3^i^	111.75 (14)	H5B—C5—H5C	109.5
F1—B1—N3^i^	111.63 (14)	C10—C6—C4	116.79 (14)
F2—B1—N2	114.07 (15)	C10—C6—C7	117.96 (15)
F1—B1—N2	112.80 (14)	C4—C6—C7	125.25 (15)
N3^i^—B1—N2	94.35 (11)	C8—C7—C6	121.57 (14)
C2—C1—H1A	109.5	C8—C7—H7	119.2
C2—C1—H1B	109.5	C6—C7—H7	119.2
H1A—C1—H1B	109.5	C7—C8—C9	118.11 (14)
C2—C1—H1C	109.5	C7—C8—H8	120.9
H1A—C1—H1C	109.5	C9—C8—H8	120.9
H1B—C1—H1C	109.5	N3—C9—N2	112.09 (13)
N1—C2—C3	122.85 (14)	N3—C9—C8	125.89 (13)
N1—C2—C1	115.96 (15)	N2—C9—C8	122.02 (15)
C3—C2—C1	121.16 (15)	N1—C10—N2	114.83 (13)
C4—C3—C2	121.17 (16)	N1—C10—C6	125.31 (15)
C4—C3—H3	119.4	N2—C10—C6	119.85 (13)
C2—C3—H3	119.4		
			
C9—N2—B1—F2	−114.73 (16)	B1^i^—N3—C9—N2	−178.24 (19)
C10—N2—B1—F2	66.2 (2)	N3^i^—N3—C9—C8	179.03 (18)
C9—N2—B1—F1	117.11 (15)	B1^i^—N3—C9—C8	1.5 (3)
C10—N2—B1—F1	−61.9 (2)	C10—N2—C9—N3	178.49 (15)
C9—N2—B1—N3^i^	1.44 (17)	B1—N2—C9—N3	−0.6 (2)
C10—N2—B1—N3^i^	−177.59 (15)	C10—N2—C9—C8	−1.3 (2)
C10—N1—C2—C3	−1.4 (2)	B1—N2—C9—C8	179.64 (15)
C10—N1—C2—C1	176.83 (15)	C7—C8—C9—N3	−177.38 (17)
N1—C2—C3—C4	2.4 (3)	C7—C8—C9—N2	2.3 (3)
C1—C2—C3—C4	−175.71 (16)	C2—N1—C10—N2	−179.60 (14)
C2—C3—C4—C6	−1.2 (3)	C2—N1—C10—C6	−0.7 (2)
C2—C3—C4—C5	177.65 (17)	C9—N2—C10—N1	178.44 (15)
C3—C4—C6—C10	−0.7 (2)	B1—N2—C10—N1	−2.6 (2)
C5—C4—C6—C10	−179.57 (16)	C9—N2—C10—C6	−0.5 (2)
C3—C4—C6—C7	178.76 (16)	B1—N2—C10—C6	178.48 (16)
C5—C4—C6—C7	−0.1 (3)	C4—C6—C10—N1	1.8 (3)
C10—C6—C7—C8	0.0 (2)	C7—C6—C10—N1	−177.73 (16)
C4—C6—C7—C8	−179.45 (17)	C4—C6—C10—N2	−179.38 (15)
C6—C7—C8—C9	−1.7 (2)	C7—C6—C10—N2	1.1 (2)
N3^i^—N3—C9—N2	−0.7 (2)		

Symmetry codes: (i) −*x*, −*y*, −*z*+2.

### Hydrogen-bond geometry (Å, °)

**Table:**

*D*—H···*A*	*D*—H	H···*A*	*D*···*A*	*D*—H···*A*
C1—H1C···F1^ii^	0.96	2.54	3.291 (2)	135
C8—H8···F1^iii^	0.93	2.48	3.2434 (19)	140

Symmetry codes: (ii) *x*+1/2, −*y*+1/2, −*z*+2; (iii) −*x*+1/2, *y*−1/2, *z*.
